# Enhancing Pulmonary Embolism Mortality Risk Stratification Using Machine Learning: The Role of the Neutrophil-to-Lymphocyte Ratio

**DOI:** 10.3390/jcm13051191

**Published:** 2024-02-20

**Authors:** Minodora Teodoru, Mihai Octavian Negrea, Andreea Cozgarea, Dragoș Cozma, Adrian Boicean

**Affiliations:** 1 Medical Clinical Department, Faculty of Medicine, “Lucian Blaga” University, 550024 Sibiu, Romania; minodora.teodoru@ulbsibiu.ro (M.T.); adrian.boicean@ulbsibiu.ro (A.B.); 2 County Clinical Emergency Hospital of Sibiu, 550245 Sibiu, Romania; andreea.cozgarea@umft.ro; 3 Institute of Cardiovascular Diseases Timisoara, 300310 Timisoara, Romania; dragos.cozma@umft.ro; 4 Cardiology Department, “Victor Babeș” University of Medicine and Pharmacy, 300041 Timisoara, Romania; 5 Research Center of the Institute of Cardiovascular Diseases Timișoara, 300310 Timisoara, Romania

**Keywords:** acute pulmonary embolism, neutrophil-to-lymphocyte ratio, in-hospital mortality, two-step cluster analysis, classification and regression trees

## Abstract

(1) Background: Acute pulmonary embolism (PE) is a significant public health concern that requires efficient risk estimation to optimize patient care and resource allocation. The purpose of this retrospective study was to show the correlation of NLR (neutrophil-to-lymphocyte ratio) and PESI (pulmonary embolism severity index)/sPESI (simplified PESI) in determining the risk of in-hospital mortality in patients with pulmonary thromboembolism. (2) Methods: A total of 160 patients admitted at the County Clinical Emergency Hospital of Sibiu from 2019 to 2022 were included and their hospital records were analyzed. (3) Results: Elevated NLR values were significantly correlated with increased in-hospital mortality. Furthermore, elevated NLR was associated with PESI and sPESI scores and their categories, as well as the individual components of these parameters, namely increasing age, hypotension, hypoxemia, and altered mental status. We leveraged the advantages of machine learning algorithms to integrate elevated NLR into PE risk stratification. Utilizing two-step cluster analysis and CART (classification and regression trees), several distinct patient subgroups emerged with varying in-hospital mortality rates based on combinations of previously validated score categories or their defining elements and elevated NLR, WBC (white blood cell) count, or the presence COVID-19 infection. (4) Conclusion: The findings suggest that integrating these parameters in risk stratification can aid in improving predictive accuracy of estimating the in-hospital mortality of PE patients.

## 1. Introduction

Venous thromboembolism (VTE), comprising pulmonary embolism (PE) and deep venous thrombosis (DVT), is the third most prevalent acute cardiovascular syndrome, after myocardial infarction and stroke [1]. VTE, with its potentially debilitating and often even fatal progression, poses a significant public health concern, particularly given its rising incidence in an aging population [2,3,4].

Accurate risk estimation in PE is of paramount importance for the efficient allocation of medical resources in an effort to enhance patient care. The pulmonary embolism severity index (PESI) stands as a prominent and validated tool for evaluating 30-day mortality risk, incorporating eleven distinct factors [5]. Additionally, a condensed version, the simplified PESI (sPESI), has been developed, demonstrating a high efficiency [6].

Other measurements include those obtained by echocardiography. While echocardiographic parameters alone may not possess high specificity or sensitivity for PE, certain measurements can suggest compromised right ventricular function. These include an enlarged right ventricle, diminished pulmonary acceleration time, reduced tricuspid annular systolic plane excursion (TAPSE), and an elevated RV/LV ratio [7,8].

The COVID-19 pandemic has notably intensified the focus on managing patients with pulmonary embolism (PE), primarily due to the hypercoagulability associated with the virus [9]. This heightened interest has also been driven by the well-established link between COVID-19 and a wide array of thrombotic complications [10,11,12]. 

Efforts to further refine risk stratification in acute PE are underway and certain parameters have shown promise in this regard. NLR, for example, has been established as a useful metric in predicting outcomes in PE patients. Elevated NLR levels have been shown to correlate with increased mortality and length of hospital stay, suggesting that NLR could augment traditional risk stratification scores like PESI and sPESI [13,14].

Furthermore, NLR has emerged as a potential prognostic marker across diverse conditions such as sepsis, pneumonia, COVID-19, and neoplastic diseases. Despite the absence of a consensus on the optimal NLR threshold, a higher value of this parameter has been recognized as an independent marker of immune system imbalance and mortality risk in both general and disease-specific cohorts [15]. At its core, the NLR is postulated to mirror the balance between acute inflammation (neutrophil count) and adaptive immunity (lymphocyte count) [16], explaining its value in the realm of chronic diseases, where inflammation and immunity play pivotal roles. Moreover, inflammation and oxidative stress are acknowledged as key contributors to the pathogenesis of cardiovascular disease, spurring extensive research into inflammatory biomarkers. Particularly, elevated NLR levels have been independently and significantly linked with a more severe prognosis in a wide range of cardiovascular afflictions, as well as increased risks of all-cause mortality, cardiovascular mortality, and mortality from other causes [17,18,19]. NLR stands out due to its cost-effectiveness, accessibility, and capability to enhance risk stratification beyond traditional scores, offering crucial insights into predicting both in-hospital and long-term mortality [20,21].

Emerging evidence underscores a significant overlap in the pathogenic mechanisms of hypercoagulability and inflammation in cases of COVID-19 and pulmonary embolism, mainly showcasing the involvement of cellular-mediated immunity and the use of NLR as a potential marker in this regard [22]. The convergence of these mechanisms is particularly relevant in the pursuit of improved mortality prediction methods. 

Although prior research has identified associations between NLR [14], COVID-19 [23], and mortality in PE, integrating these factors with established clinical scores has not been sufficiently explored. The purpose of the present study was to show the correlation of NLR and PESI/SPESI in determining the risk of in-hospital mortality in patients with pulmonary thromboembolism. In addition, we sought to investigate the impact of COVID-19 infection and other parameters on the aforementioned outcome. By employing machine learning techniques, specifically two-step cluster analysis and classification and regression trees, we aimed to refine patient risk stratification and enhance the predictive accuracy of existing tools. 

## 2. Materials and Methods

### 2.1. Study Design and Data Collection

We conducted a retrospective analysis of data extracted from the hospital records of 160 patients admitted to the County Clinical Emergency Hospital of Sibiu diagnosed with acute pulmonary embolism between January 2019 and December 2021. To achieve this, the records were searched within the primary and secondary diagnoses fields for the diagnosis codes corresponding to acute pulmonary embolism according to the International Classification of Disease-ICD-10 (I26.0; I26.9). Only entries containing acute events were included in the study; i.e., instances where the aforementioned diagnosis codes referred to previous events in the patient’s history were excluded. Diagnostic criteria were checked according to the most recent guidelines published by the European Society of Cardiology (ESC) on the diagnosis and management of acute pulmonary embolism [8] and relied mainly on imaging confirmation of pulmonary embolism via computed tomography pulmonary angiography. 

Characteristics describing patient demographics (age, gender), medical history, and clinical presentation (including vital parameters on arrival), as well as laboratory and imaging findings were extracted. PESI and SPESI scores were subsequently calculated retrospectively according to the instructions in the guidelines mentioned above. Retrospective computation of these scores has been validated in previous, more extensive retrospective cohort studies, providing reliable results [24]. Classification into risk classes according to PESI and sPESI risk scores was implemented using the following cut-offs, as endorsed by the same guidelines published by the ESC [8]:

- PESI:
  ○ Very low risk: ≤65 points
  ○ Low risk: 66–85 points
  ○ Intermediate risk: 86–105 points.
  ○ High risk: 106–125 points
  ○ Very high risk: >125 points
- sPESI
  ○ Low risk: 0 points
  ○ High risk: ≥1 point(s)

In addition, the presence or absence of concomitant deep vein thrombosis was noted, as well as the presence of COVID-19 infection either on admission or in the 14 days prior to the patient’s presentation as documented according to local hospital protocols, which were implemented during the first wave of the COVID-19 pandemic. The neutrophil-to-lymphocyte ratio was computed from the first available blood sample drawn within the first 14 days of hospital admission. Due to the known variability in NLR over time, patients were stratified according to the timeframe within which the first complete blood count was available, and a subanalysis of patients with a CBC available in the first 24 h of PE diagnosis was performed. Analysis of NLR values recorded in the first 24 h after PE diagnoses is a similar approach to the one implemented by Efros et al. [14]. Patients with no available blood samples were excluded from the study. 

Blood tests were performed after venous blood sample collection. CBCs, including total white blood cell, neutrophil, and lymphocyte counts, were computed using fluorescent flow cytometry on an automatic hematology analyzer. NLR was calculated as the ratio between the absolute number of neutrophil granulocytes and the absolute number of lymphocytes, as described previously [16,17]. Leukocytosis was defined as a WBC (white blood cell) count above 10 × 10^3^/µL, similarly to Afzal et al. [25]. Variables that presented missing data within the study group were excluded from the analysis. Among other determinations, this included C-reactive protein levels, which were not routinely measured.

Echocardiographic data were also extracted, as all the patients admitted with the diagnosis of PE had undergone echocardiographic evaluation at the time of diagnosis. The presence of dilated right ventricle (parasternal long axis proximal RVOT diameter above 30 mm), altered right ventricle function (tricuspid annular plane systolic excursion under 16 mm), dilated inferior vena cava (above 20 mm), or the presence intracavitary thrombus were documented. These measurements and their cut-offs were based on the current recommendations published by the European Society of Cardiology (ESC) on the diagnosis and management of acute pulmonary embolism [8] and current guidelines on echocardiographic chamber quantification [26].

### 2.2. Data Processing

Elevated NLR was defined similarly to Efros et al. [14], whereby, in the absence of a unanimously accepted cut-off value for NLR for predicting PE outcomes, patients with an NLR above the median of the collected sample were compared to those with values below this value, essentially providing a dichotomous variable in this regard. Consequently, patient stratification according to the timeframe of blood sample collection (i.e., within the first 24 h of PE diagnosis vs. all patients, regardless of the moment in which the first CBC was acquired within the first 14 days of hospital admission) yielded different cut-offs in our stratified analysis. Our primary outcome variable was in-hospital mortality. Statistical analysis was executed utilizing the IBM SPSS Statistics 21 software package. Numerical variables were described by their mean, median, standard deviation, 95% confidence interval for the mean, minimum, maximum, and interquartile range values. To evaluate the normality of continuous variables, the Shapiro–Wilk and Kolmogorov–Smirnoff tests were utilized where appropriate, together with the evaluation of the skewness and kurtosis of the data. Categorical variables were described by computing their frequency distribution. 

#### 2.2.1. Bivariate Analysis

For continuous variables conforming to a normal distribution, t-Student tests were applied for comparative analysis. Otherwise, a Mann–Whitney U test was implemented. Chi-square or Fisher exact tests were used to identify significant associations between categorical variables. A *p*-value less than 0.05 was regarded as indicative of statistical significance.

#### 2.2.2. Multivariate Logistic Regression and ROC Curve Comparison

In order to quantify the impact of each predictor for in-hospital mortality identified in the initial bivariate analysis, binary logistic regression was performed. 

The methodology employed involved iterative inclusion or exclusion of variables to identify the best-fitting regression model. Numerical variables were mean-centered to mitigate multicollinearity, while categorical variables were transformed into dummy variables. Bootstrapping with 1000 samples was performed to determine 95% confidence intervals for the regression coefficients using the bias-corrected and accelerated (BCa) method. Variables that significantly influenced in-hospital mortality prediction (*p* < 0.05 and both limits of the 95% confidence interval for coefficients being positive) were retained in the model.

In addition, ROC curves were computed for numerical variables, in order to further illustrate their comparative accuracy in predicting in-hospital mortality.

#### 2.2.3. Machine Learning Algorithms

To further enhance our findings, we employed two machine learning methods, namely a two-step cluster analysis and a classification and regression tree algorithm. This approach was undertaken to discern complex patterns and relationships within the dataset. In our iterative process, variables demonstrating significant correlations with in-hospital mortality were systematically incorporated and subsequently eliminated from the models, aiming to identify the most effective combinations of variables that could reliably predict our target outcome. 

Two-step clustering used the k-means algorithm and hierarchical agglomerative clustering to delineate groups of patients with similar characteristics regarding the variables employed. While this is an unsupervised method, by feeding the algorithm with variables that correlate with a specific outcome (in our case, in-hospital mortality), the traits of each resulting cluster can converge with respect to this outcome, yielding distinct populations in this regard. We used Akaike’s information criterion (AIC) to determine the optimal model fit and allowed for automatic selection for the number of clusters. We selected models with an average silhouette of cohesion separation of at least 0.5 to indicate their robustness. In addition, variables with a predictor importance under 0.5 were discarded from the models to enhance their quality.

CART decision trees also delineate between different patient groups based on specified characteristics. This technique is, however, supervised, whereby the outcome variable is predefined, thus enabling such algorithms to provide prediction models for the investigated outcome. In addition, it delivers a visual model to illustrate the complex interplay between predictors and outcomes, without attempting, however, to provide a causal explanation for the defined rule set.

The construction of the model is executed from the primary root and expands through branching until further division is no longer feasible, correlating all predictors to anticipate the investigated outcome (in our case, in-hospital mortality). Branching is guided by conditions (internal nodes) imposed on predictor variables, which iteratively segment the data. The endpoint of a branch (referred to as a “leaf” or child node) signifies the conclusive decision of the algorithm. The defining parameters involved in tree growth in CART decision trees are based on the principle of entropy, whereby data segmentation across nodes is governed by the reduction in node impurity from one split to the next. The primary objective is to pinpoint the optimal split point (cut-off value) for a predictor variable. Division criteria are optimized based on the Gini index and the Twoing impurity metrics for categorical variables or the LSD (least squares deviation) impurity measure for continuous variables. The algorithm then ascertains the best node division by choosing the predictor that optimizes the division criterion, culminating in the maximal decrease in node impurity, repeating the process of each “child” node until no further enhancement is feasible or pre-established stopping criteria are met. The CART decision tree is characterized by its adaptability for managing various data types and distributions, resilience against outliers, and efficient treatment of missing values through surrogate divisions. 

Following the tree’s full expansion, pruning trims the tree (eliminating nodes that contribute minimal additional information) to the most compact subtree with an acceptable risk level. This mitigates the risk of overfitting the model to the input data and enhances its stability.

In this study, CART models were computed in pruning mode, considering variables that correlated with in-hospital mortality. To grow the decision tree model, we allowed for automatic selection of maximum growth levels (5 by default), with 5 as the minimum number of cases for parent nodes and 3 for child nodes. For the Gini impurity measure, we selected a minimum change in improvement of 0.0001, and the maximum difference in risk in standard errors was set to 0. 

Both techniques are adept at analyzing continuous and categorical variables, despite employing different underlying mathematical constructs. In addition, they have demonstrated their usefulness in enhancing insights from clinical data, even in small sample sizes. Due to the different approaches of the two algorithms towards classifying data, the results they yield are complementary to each other, offering valuable perspectives on patient categorization. These aspects were described in more detail in our previous work [27].

## 3. Results

### 3.1. Bivariate Analysis

There were 160 patients included in our study, 76 (47.5%) of whom were female and 84 (52.5%) male. Table 1 shows the distribution of patients according to the first available CBC timeframe.

Table 2 and Table 3 show the characteristics of the studied group across genders. No cases with a body temperature under 36 °C were recorded within our study group. Median NLR was 3.7 when considering all patients and 4.69 when analyzing the subgroup of patients with a CBC available within the first 24 h of PE diagnosis.

Patients with NLR values above the median were categorized as having elevated NLR. Patients with a CBC available within the first 24 h of PE diagnosis were recategorized according to the median of their group when subanalyzed.

Table 4 and Table 5 provide information on the distribution of variables across NLR categories.

Table 6 and Table 7 exhibit the distribution of variables in reference to in-hospital mortality.

### 3.2. Multivariate Binary Logistic Regression and ROC Curves

Four numerical and ten categorical variables showed significant correlations with in-hospital mortality within the entire group. WBC count and the presence of chronic heart failure, chronic pulmonary disease, or a respiratory rate above 30 breaths/min on admission correlated with mortality when considering the entire group, but not within the <24 h CBC subanalysis. Binary regression was performed to identify the strongest predictors for in-hospital mortality. When analyzing the group as a whole, an adequate binary logistic regression model for predicting in-hospital mortality was obtained when retaining the variables defining the presence of COVID-19 infection, elevated NLR, and altered mental status or hypoxemia on admission. 

The model had an overall efficiency of 90.6% (57.7% for predicting in-hospital mortality and 97% for predicting survival) and satisfactory goodness of fit (Hosmer–Lemeshow *p*-value = 0.589). The results containing the statistical significance of the selected variables and the 95% confidence intervals for the regression coefficients calculated via the BCa method are presented in Table 8.

**Figure 1:**
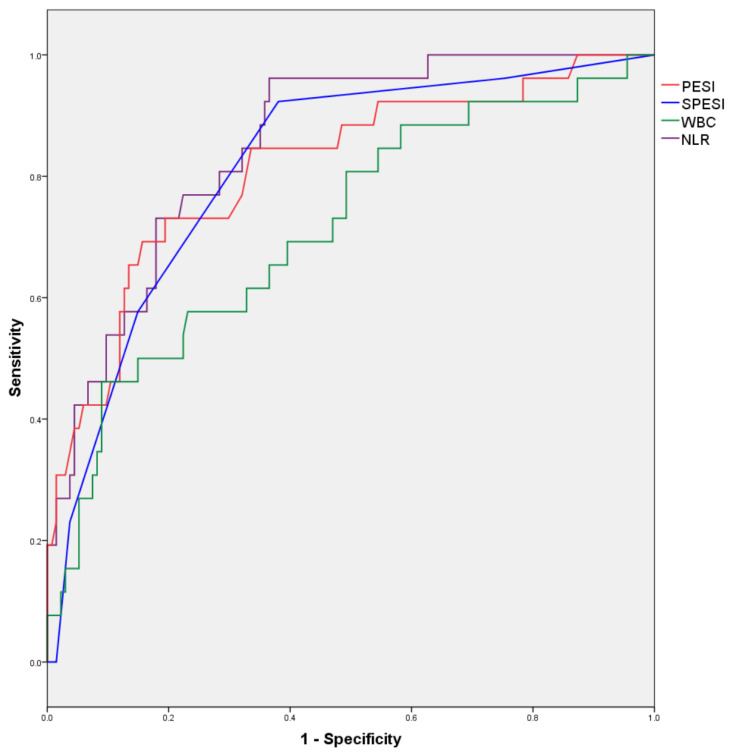
ROC curves for numerical variables predicting in-hospital mortality.

ROC curves for numerical variables found to correlate with in-hospital mortality in the bivariate analysis are illustrated in Figure 1, and the areas under the resulting ROC curves are presented in Table 9.

Areas under the ROC curves are displayed in Table 9.

**Figure 2:**
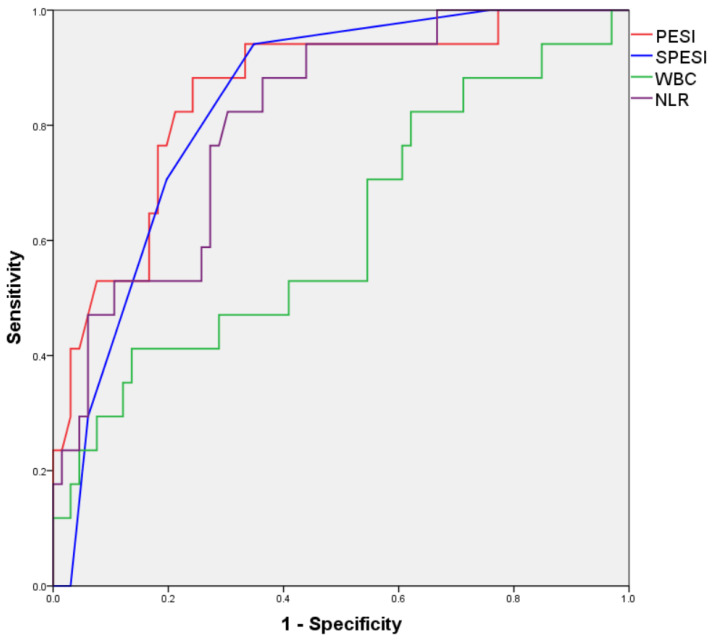
ROC curves for numerical variables predicting in-hospital mortality (<24 h CBC subanalysis).

A subanalysis of the group with a CBC available in the first 24 h after PE diagnosis was also performed; however, an adequate binary logistic regression model for predicting in-hospital mortality could not be obtained. ROC curves for numerical variables found to correlate with in-hospital mortality in bivariate analysis are illustrated in Figure 2 and the areas under the resulting ROC curves are presented in Table 10.

### 3.3. Two-Step Cluster Analysis

We performed a two-step cluster analysis to enhance the understanding of the interplay between traditional risk scores, the presence of COVID-19 infection, and NLR values.

**Figure 3:**
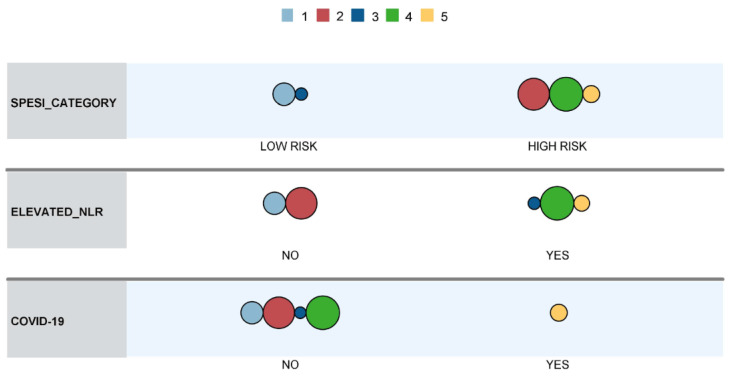
Cluster comparison.

Of the tested models, a robust variant with an average silhouette of cohesion separation of approximately 1.0 was obtained by using the sPESI category, NLR category (i.e., above or below median), and COVID-19 coinfection. The distribution of variables within the model and its clusters is presented in Table 11, and a visual representation of the model is illustrated in Figure 3.

**Figure 4:**
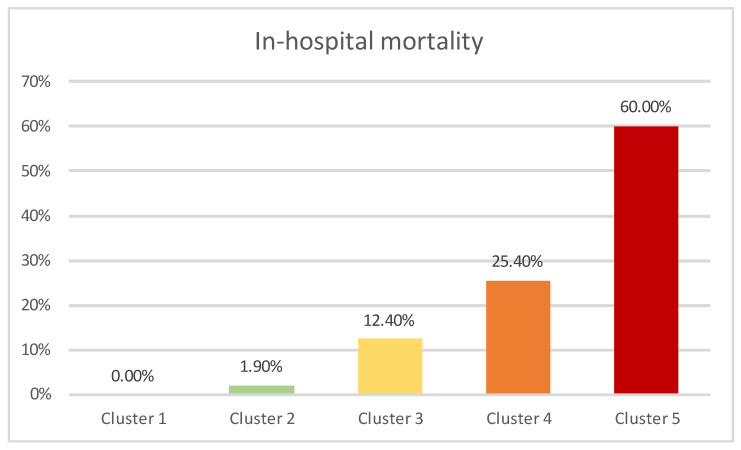
In-hospital mortality across clusters.

The frequency of in-hospital mortality across resulting clusters is presented in Figure 4. The differences observed were statistically significant (*p* < 0.01).

Cluster 5, with the highest mortality, was exclusively comprised of COVID-19 patients, who were classified into the high-risk sPESI category and had elevated NLR. Clusters 1–4 were mainly composed of non-COVID-19 patients (a single case in cluster 3). Cluster 4 contained patients classified into the high-risk sPESI category, which had elevated NLR, while patients in cluster 1 (which showed the lowest in-hospital mortality) were categorized as low risk according to the sPESI score and did not have elevated NLR values. Cluster 2 contained patients classified as high risk according to sPESI score without having elevated NLR values, while Cluster 3 was comprised of patients categorized as low-risk according to sPESI score but who had elevated NLR values.

When analyzing the subgroup of patients with a CBC available in the first 24 h after admission, two-step cluster analysis based on the same variables yielded a model containing only four clusters, with an average silhouette of cohesion separation of 0.9. The distribution of variables within the model and its clusters is presented in Table 12.

**Figure 5:**
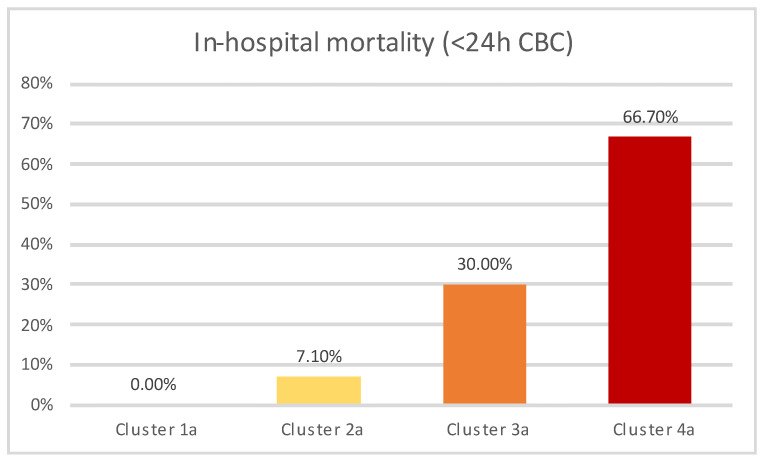
In-hospital mortality across clusters (<24 h CBC subanalysis).

The frequency of in-hospital mortality across resulting clusters is presented in Figure 5, with the differences being statistically significant (*p* < 0.01).

Similar clustering tendencies were observed in the subanalysis, with one cluster comprised exclusively of COVID-19 patients (Cluster 4a) classified as high-risk according to SPESI score, while the rest of the clusters (Clusters 1a–3a) were composed of non-COVID-19 patients. Cluster 3 contained patients who were both classified as high-risk sPESI category and additionally presented elevated NLR levels, while patients in cluster 1a (which showed the lowest in-hospital mortality) were all categorized as low-risk according to the sPESI score, in addition to most frequently not having elevated NLR values. Cluster 2a contained patients categorized as high-risk according to SPESI, while not exhibiting elevated NLR levels, and showed an intermediary value between cluster 1a and 3a regarding in-hospital mortality.

### 3.4. CART Decision Tree

**Figure 6:**
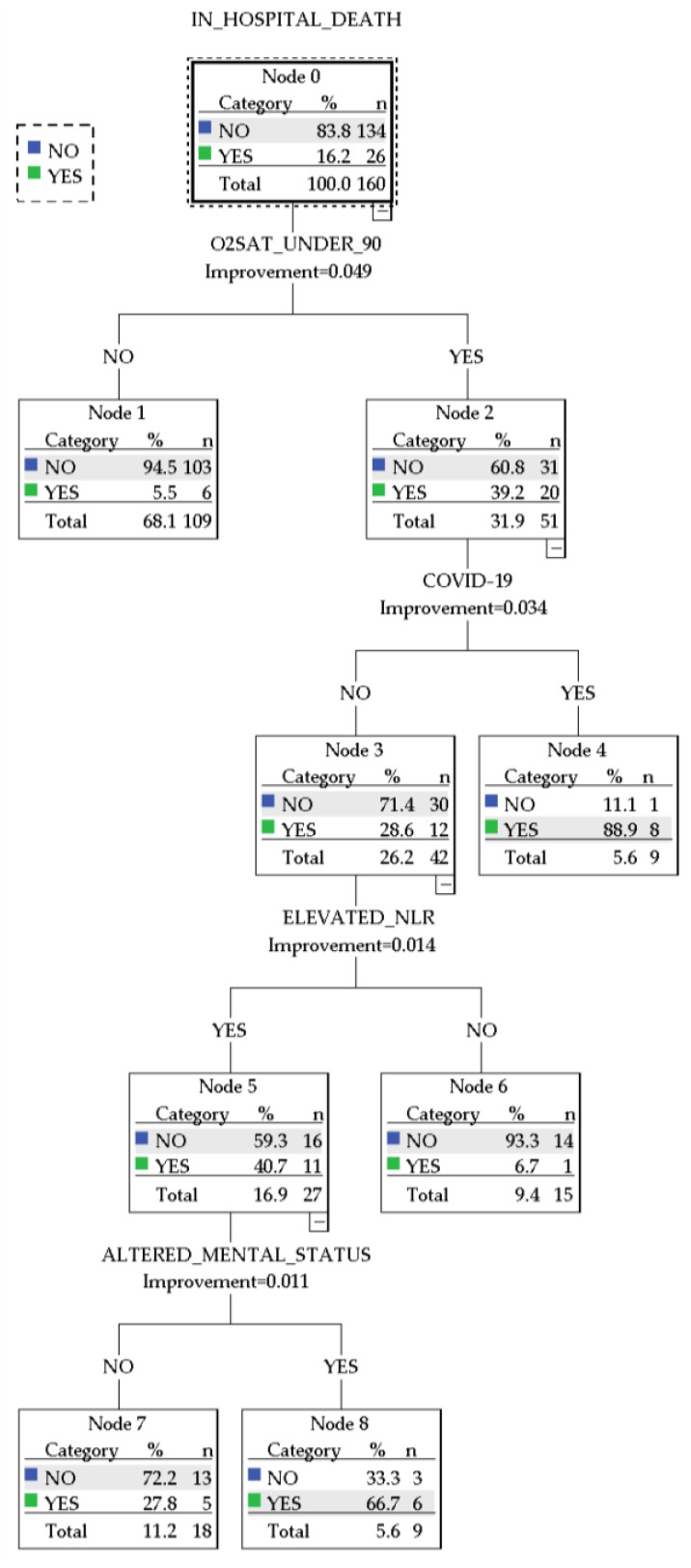
CART decision tree.

A cart decision tree was generated using the following variables: the presence of COVID-19 infection on admission or in the 14 days prior, arterial oxyhemoglobin saturation <90%, the presence of altered mental status, and the presence of NLR above the median. The resulting model is presented in Figure 6.

The CART decision tree showed an overall accuracy of 90% (97.3% for predicting survival and 53.8% for predicting in-hospital death). The decision paths in the algorithm distinguished between several patient groups with distinct characteristics regarding the presence of COVID-19 infection, elevated NLR, and particular definitory elements of the PESI/sPESI scores. Notably, the following patient subgroups emerged:

- A group of 9 patients with arterial oxyhemoglobin saturation <90% infected with COVID-19, with an 88.9% prediction chance of in-hospital mortality
- A group of 9 patients without COVID-19 presented with altered mental status, arterial oxyhemoglobin saturation <90%, and elevated NLR and had a 66.7% prediction chance of in-hospital mortality.
- A group of 18 patients without COVID-19 who presented with arterial oxyhemoglobin saturation <90% and elevated NLR and had a 27.8% prediction chance of in-hospital mortality
- A group of 15 patients without COVID-19 who presented with arterial oxyhemoglobin saturation <90% but did not have elevated NLR. These patients had a 6.7% chance of predicted in-hospital mortality.
- A group of 109 patients who presented with normal arterial oxyhemoglobin saturation. These patients were not further stratified and had a 5.5% predicted chance of in-hospital mortality.

**Figure 7:**
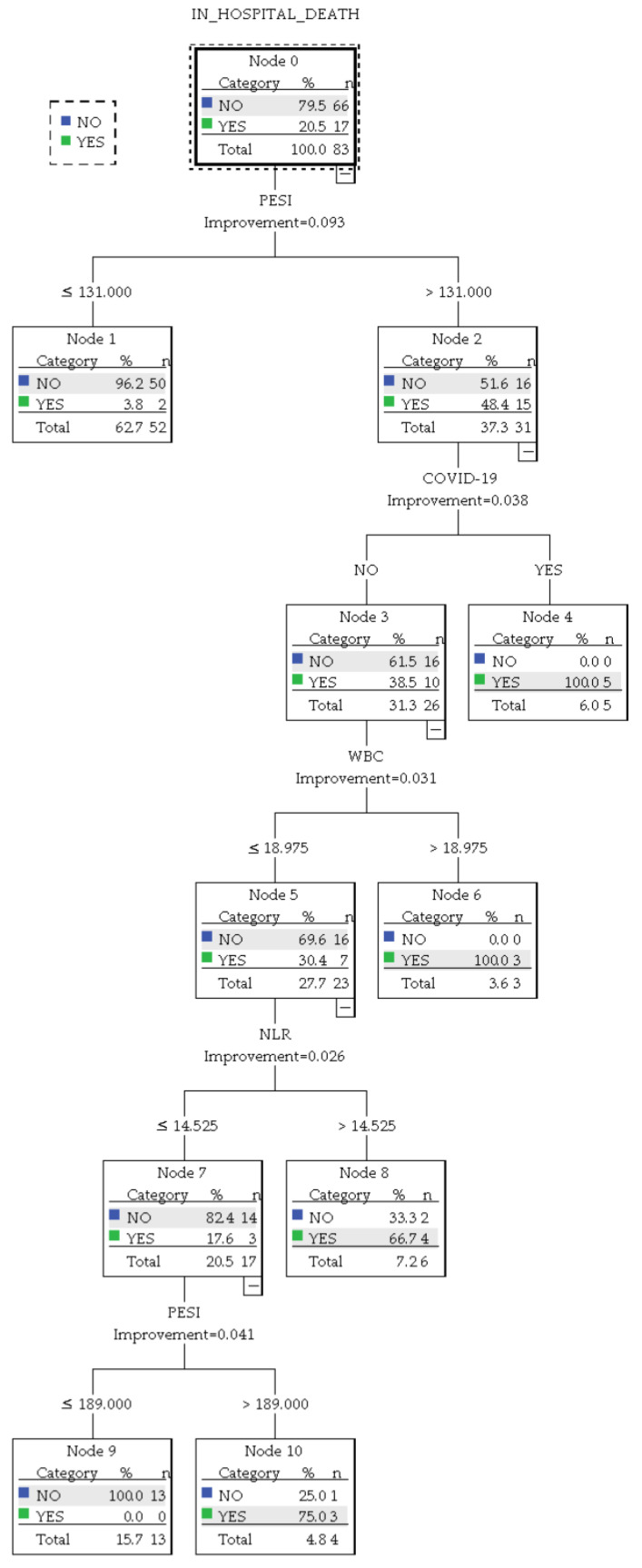
CART decision tree (<24 h CBC subanalysis).

When analyzing the subgroup of patients with a CBC available in the first 24 h, compared to the whole group analysis, a more robust model was obtained when implementing mostly numerical variables concerning traditional PE risk estimation strategies. The result is presented in Figure 7.

This iteration delivered an overall accuracy of 94% (95.5% for predicting survival and 88.2% for predicting in-hospital death). Based on the presence of COVID-19 infection, NLR levels, and PESI score, the algorithm identified the following patient subgroups:

- A group of 5 patients infected with COVID-19 and a PESI score above 131 with a 100% prediction chance of in-hospital mortality.
- A group of 3 patients without COVID-19 who presented with a WBC count above 18.975 × 10^3^/µL and an NLR above 14.525 with a 100% prediction chance of in-hospital mortality.
- A group of 4 patients without COVID-19 who had a WBC count up to 18.975 × 10^3^/µL and an NLR up to 14.525 but presented a PESI score above 189. In this group, the predicted chance of in-hospital mortality was 75%.
- A group of 6 patients without COVID-19 and with a WBC up to 18.975 × 10^3^/µL but with a PESI score above 131 and an NLR above 14.525. This group was predicted to have a 66.7% chance of in-hospital mortality.
- A group of 52 patients with a PESI score under 131, who had a 3.8% chance of in-hospital mortality.
- A group of 13 patients with a PESI score between 131 and 189, who had a WBC count up to 18.975 × 10^3^/µL and an NLR up to 14.525. This group had a 0% predicted chance of in-hospital mortality.

## 4. Discussion

We conducted a retrospective analysis of 160 patients presenting with acute pulmonary embolism to investigate the significance of NLR concerning in-hospital mortality and its correlation with established prognostic tools, particularly PESI and sPESI scores.

In our study group, males were more susceptible to malignancies and chronic pulmonary diseases. These findings are in agreement with previously described results [28,29]. The inclusion of gender-based analysis in our study stemmed from recognized differences in pulmonary embolism (PE) presentation, risk factors, and outcomes between genders, as substantiated by the existing literature [30,31]. Acknowledging gender as a potential confounding factor, we aimed to ensure the comprehensive applicability of our findings across both genders.

Elevated NLR, defined by values above the median of the studied group, was significantly associated with a wide array of characteristics correlated with poor prognosis in pulmonary embolism. Importantly, our data demonstrated statistical significance in the association between elevated NLR and in-hospital mortality, as well as higher PESI and sPESI scores. This finding reinforces the previously described results, which support the idea that a high NLR is a reliable predictor of mortality in pulmonary embolism [32]. Moreover, elevated NLR showed significant correlations with a series of individual parameters used in the PESI and sPESI scores, known to influence outcomes independently in PE [33]. Namely, more advanced age, the presence of neoplasms, arterial hypotension, altered mental status, and oxygen desaturation were associated with elevated NLR. Similar findings have been reported in the literature concerning the link between NLR and age [19] and with cancer [34].

During the COVID-19 pandemic, NLR gained recognition for its potential to identify immune and inflammatory imbalances. Though preliminary in nature, due to the small sample size (16 COVID-19 patients), our data indicated a significant correlation between elevated NLR and COVID-19 infection. Both of these entities have been linked to increased mortality rates among hospitalized patients [35].

The association between COVID-19 and pulmonary embolism has been a subject of considerable attention in previous research [36,37] and the deleterious impact of COVID-19 infection on the mortality of PE patients has been thoroughly documented [23]. Our study showed similar correlations.

PESI and sPESI scores were, as anticipated, significantly increased in patients who experienced a fatal outcome. With regards to the individual elements of the PESI/sPESI scores, chronic heart failure, chronic pulmonary disease, arterial hypotension, tachypnoea, altered mental status, and hypoxemia were all correlated with increased in-hospital mortality, as also described in the original article by Aujesky et al. that first defined the PESI score [33].

To explore the specific role of NLR in risk stratification for in-hospital mortality, we utilized a range of machine learning algorithms as a novel methodology in this area of study. 

Our two-step cluster analysis yielded a highly robust model that categorized patients based on the presence of COVID-19, sPESI classification, and elevated NLR. This model delineated distinct clusters with significantly disparate in-hospital mortality rates. Notably, COVID-19 emerged as a differentiating factor, identifying a subset within cluster 5, which contained nearly all the COVID-19-positive patients of our study group and had a mortality of 60%. In addition, they displayed elevated NLR, and most were classified as high-risk according to sPESI. In the remaining patient groups, the cluster characterized by both increased NLR and high-risk sPESI (cluster 4) exhibited the highest mortality rate. In contrast, patients with low NLR and low-risk sPESI class (Cluster 1) experienced a 0% mortality rate. The transition to clusters 2 and 3 underscores the potential modulatory effect of NLR on risk stratification. Despite cluster 3 patients being classified as low-risk according to sPESI, they exhibited more frequent elevated NLR levels and were associated with a significantly higher mortality rate compared to patients in cluster 2, who were deemed high-risk based on sPESI criteria. It is important to note that although cluster 3 included a COVID-19-positive patient, this individual did not succumb to the illness, suggesting that factors other than COVID-19 status, such as elevated NLR, maintain their validity in mortality prediction.

The CART algorithm further nuanced the role of elevated NLR in this regard, showing an overall accuracy of 90% based on hypoxemia, the presence of COVID-19 infection, elevated NLR, and altered mental status, while offering a visual framework in this regard. The algorithm’s performance was particularly high in predicting survival (97.3%), while showing a more modest performance for in-hospital death prediction (53.8%). This discrepancy highlights the potential utility of the algorithm in developing screening tools that could expediently stratify patients, particularly low-risk individuals, utilizing readily available data.

The decision tree identified oxygen saturation below 90% as the primary stratifying factor, significantly correlating with increased in-hospital mortality rates. Subsequent bifurcations in the tree revealed that the presence of COVID-19 infection may influence the risk of in-hospital mortality. Further divisions within the tree highlighted elevated NLR as a modulatory factor, suggesting its utility as a prognostic marker in the hierarchical assessment of patient risk. The recognition of elevated NLR as a significant predictor of mortality invites further investigation into its pathophysiological roles and potential integration into comprehensive risk assessment models. Ultimately, this decision tree provides a data-driven approach model for prioritizing clinical interventions resource allocation.

To address potential variations in NLR measurements due to timing, a focused subanalysis was conducted on patients with a complete blood count (CBC) obtained within the first 24 h after PE diagnosis. This timeframe has been previously validated in the literature, exploring the significance of NLR in PE prognosis estimation [14]. The subanalysis recalibrated the elevated NLR threshold based on the median of this subset, revealing a minor deviation in the cut-off value (4.69 compared to the initial 3.7). While slight variations in the correlation between elevated NLR and PE prognostic factors were noted, the fundamental prognostic significance of NLR, particularly in its association with in-hospital mortality when integrated with conventional risk scores, was reaffirmed.

In addition, cluster analysis revealed consistent patient stratification patterns, distinguishing COVID-19 patients automatically and highlighting their distinct prognosis in the entire group and also in the subanalyzed group. The clustering of non-COVID-19 patients according to SPESI category and elevated NLR showed similar patterns with the analysis of the whole group, iteratively showcasing the modulatory impact of NLR on patient prognosis, whereby mortality differed significantly across clusters in a consistent manner across the analyzed study groups.

The CART-based algorithm, however, displayed enhanced precision with numerical variables in the subanalysis, while in the analysis of the entire group, the use of categorical variables yielded a satisfactory model. Despite employing an adjusted NLR threshold for patient sub-stratification, this algorithm nonetheless remained consistent in illustrating the overall predictive relevance of NLR for in-hospital mortality across both the complete and subanalyzed groups.

### Strengths and Limitations

The current study utilized well-established prognostic tools, notably PESI and sPESI scores, which have been critically validated for evaluating mortality risk. This alignment with clinical standards underpins the methodological robustness of the research. Incorporating advanced machine learning techniques, such as two-step cluster analysis and classification and regression trees (CART), introduces a novel element to the field. These techniques offer new insights into complex patterns that may not be apparent through traditional statistical methods, offering significant advantages in pathologies that are influenced by a wide array of variables, such as pulmonary embolism. The analysis of the interplay between diverse parameters, focusing on NLR and COVID-19 status, provides comprehensive risk stratification insights relevant to clinical practice, highlighting potential avenues for optimizing patient care and resource allocation.

While this study provides valuable insights into the prognostic utility of NLR and its integration with PESI/sPESI scores in the context of pulmonary embolism, particularly during the COVID-19 pandemic, we must acknowledge its limitations. The retrospective, single-center design may limit the generalizability of our findings. Data collected retrospectively can introduce biases that prospectively designed studies might avoid, such as selection bias and information bias. Our findings are reflective of a single institution’s patient population and practices, which may not be representative of broader clinical settings. In addition to the study’s reliance on data from a single center, the absence of randomization is a further limitation to be considered, which may hinder the generalizability of our findings. Furthermore, the relatively modest sample size constrained our ability to detect smaller effect sizes and may limit the statistical power of our analyses. Future studies should aim to validate our findings through multicentric, prospective research designs, which could provide a more diverse patient population and reduce potential institutional biases. Additionally, larger sample sizes would enhance the reliability of the machine learning models developed and provide a more robust predictive framework for clinical use.

Notwithstanding, the consistency of the results with findings from previous research provides a measure of validation, lending credibility to the data presented. In addition, the novel approaches described can serve as a framework for future larger studies spanning across multiple centers that could make use of randomized sample selection and prospective data collection.

A notable limitation is the undefined optimal threshold for NLR, which, in this study, was based on the sample’s median. While practical, this may not be the optimal threshold for broader patient populations and different clinical environments. However, this approach aligns with methodologies from other large-scale studies that have yielded significant results [14]. The NLR thresholds used in this study (i.e., 3.7; 4.69) were relatively close to the range of cut-off values identified in the literature. In particular, one meta-analysis mentioned NLR cut-off values for mortality prediction in PE varying between 5.4 and 9.2 [38]. The split identified by the CART algorithm, however, in the subanalysis of patients with a CBC available in the 24 h after PE diagnosis (14.525), further raised concerns regarding the optimal interpretation of this parameter, particularly in the context of time-sensitivity. Furthermore, more extensive studies should explore the impact of this parameter’s variation, as well as its definitive cut-off points for predicting cardiovascular outcomes.

Additionally, while the use of machine learning algorithms is innovative, there is a risk of overfitting the models to the particular dataset, which could reduce their predictive accuracy when applied to other populations. We mitigated this risk with the CART algorithm, however, by pruning the resulting trees. 

## 5. Conclusions

The current study presents a novel contribution to pulmonary embolism risk stratification by incorporating advanced machine learning techniques, which have elucidated complex patterns in patient data, particularly emphasizing the prognostic significance of elevated NLR in PE patients. While the study was retrospective in nature and based on data from a single center, the findings underscore the additive value of NLR in enhancing the predictive accuracy of existing tools in pulmonary embolism, while providing a nuanced perspective on patient risk assessment. Our results emphasize the possibility of refining risk prediction in PE based on NLR values, as well as additional parameters such as WBC count and COVID-19 infection status, setting a precedent for future studies to build upon its findings and methodologies.

## Figures and Tables

**Table 1:** First available CBC timeframe.

First Available CBC	Total (% of Grand Total)	Gender (% of Category)		*p*-Value
		Female	Male	
<24 h	83 (51.88%)	39 (46.99%)	44 (53.01%)	0.973
24–48 h	18 (11.25%)	9 (50%)	9 (50%)	
>48 h	59 (36.88%)	28 (47.56%)	31 (52.54%)	

**Table 2:** Patient characteristics across genders (numerical variables).

Variable	Descriptive Parameter	Whole Group			CBC < 24 h		
		Gender		*p*-Value	Gender		*p*-Value
		Female	Male		Female	Male	
Age	Mean	67.08	65.77	0.781	68.08	67.75	0.1
	StdDev	15.54	15.13		16.68	16	
	IQR	15	17		26	21	
	MIN	21	26		21	26	
	MAX	94	89		94	89	
	95% CI	63.53–70.63	62.49–69.06		62.67–73.48	57.89–67.61	
PESI	Mean	108.72	115.1	0.246	117.69	122.05	0.690
	StdDev	46.75	41.98		53.85	45.15	
	IQR	59.5	49.25		92	61.75	
	MIN	21	38		21	50	
	MAX	220	247		220	247	
	95% CI	98.04—119.4	106—24.22		100.24–135.15	108.31–135.77	
SPESI	Mean	1.55	1.56	0.823	1.67	1.75	0.625
	StdDev	1.28	1.15		1.46	1.24	
	IQR	2	1		3	2	
	MIN	0	0		0	0	
	MAX	5	5		5	5	
	95% CI	1.26—1.85	1.31—1.81		1.19–2.14	1.37–2.13	
WBC Count (10^3^/µL)	Mean	10.24	9.66	0.798	10.86	10.23	0.985
	StdDev	5.32	3.58		6.27	4.07	
	IQR	6.17	5.32		5.74	6.54	
	MIN	2.57	3.76		3.66	4.49	
	MAX	37.06	19.37		37.06	19.37	
	95% CI	8.88–12.99	8.89–10.44		8.83–12.9	9–11.48	
NLR	Mean	5.92	6.88	0.727	7.29	7.94	1
	StdDev	6.51	7.83		7.9	8.54	
	IQR	4.33	5.38		6.01	7.17	
	MIN	0.30	0.55		0.45	1.45	
	MAX	45.87	43.23		45.87	43.23	
	95% CI	4.43—7.41	5.17—8.57		4.73—9.85	5.34—10.54	

CBC < 24 h—group with first available CBC under 24 h; StdDev—standard deviation; IQR—interquartile range; MIN—minimum observed value; MAX—maximum observed value; 95% CI—95% confidence interval for the mean; PESI—pulmonary embolism severity index; SPESI—simplified pulmonary embolism severity index; WBC—white blood cell; NLR—neutrophil-to-lymphocyte ratio.

**Table 3:** Patient characteristics across genders (categorical variables, % of column categories).

Variable	Whole Group			CBC < 24 h		
	Gender		*p*-Value	Gender		*p*-Value
	Female	Male		Female	Male	
COVID-19 (positive)	4 (5.3%)	12 (14.3%)	0.057	2 (5.1%)	7 (15.9%)	0.163
Cancer	28 (36.8%)	18 (21.4%)	0.031	13 (33.3%)	16 (34.1%)	0.942
Chronic heart failure	20 (26.3%)	26 (31%)	0.518	10 (25.6%)	14 (31.8%)	0.536
Chronic pulmonary disease	12 (15.8%)	28 (33.3%)	0.01	7 (17.9%)	16 (36.4%)	0.061
Pulse rate ≥ 110 bpm	15 (19.7%)	18 (21.4%)	0.792	9 (23.1%)	11 (25%)	0.838
Systolic BP < 100 mmHg	10 (13.2%)	13 (15.5%)	0.676	9 (23.1%)	8 (18.2%)	0.581
Respiratory rate > 30 breaths/min	3 (3.9%)	8 (9.5%)	0.164	3 (7.7%)	5 (11.4%)	0.572
Altered mental status	11 (14.5%)	15 (17.9%)	0.562	7 (17.9%)	10 (22.7%)	0.590
Arterial oxyhemoglobin Saturation < 90%	24 (31.6%)	27 (32.1%)	0.939	13 (33.3%)	16 (36.4%)	0.773
Elevated NLR	40 (52.6%)	40 (47.6%)	0.527	24 (61.5%)	25 (56.8%)	0.663
Leukocytosis	32 (42.1%)	31 (36.9%)	0.501	18 (46.2%)	19 (43.2%)	0.786
Concomitant DVT	28 (36.8%)	32 (38.1%)	0.870	13 (33.3%)	15 (34.1%)	0.942
Dilated RV	32 (42.1%)	53 (63.1%)	<0.01	19 (48.7%)	26 (59.1%)	0.344
RV dysfunction	3 (3.9%)	3 (3.6%)	0.9	1 (2.6%)	2 (4.5%)	1
Dilated VCI	5 (6.6%)	9 (10.7%)	0.355	2 (5.1%)	4 (9.1%)	0.679
Intracavitary thrombus	3 (3.9%)	2 (2.4%)	0.570	2 (5.1%)	0 (0%)	0.218
In-hospital death	13 (17.1%)	13 (15.5%)	0.780	8 (20.5%)	9 (20.5%)	0.995

BP—blood pressure; NLR—neutrophil-to-lymphocyte ratio; DVT—deep vein thrombosis; RV—right ventricle; VCI—vena cava inferior.

**Table 4:** Patient characteristics across NLR categories (numerical variables).

Variable	Descriptive Parameter	Whole Group			CBC < 24 h		
		Elevated NLR (>3.7)		*p*-Value	Elevated NLR (>4.69)		*p*-Value
		No	Yes		No	Yes	
Age	Mean	62.53	70.26	<0.01	61.4	69.2	0.03
	StdDev	16.53	12.92		16.49	15.61	
	IQR	22	15		25	20	
	MIN	21	28		21	28	
	MAX	86	94		87	94	
	95% CI	58.85–66.2	67.39–73.14		56.27—66.54	64.27—74.12	
WBC Count (10^3^/µL)	Mean	8.16	11.71	<0.01	8.38	12.74	<0.01
	StdDev	3.32	4.81		3.72	5.56	
	IQR	3.06	5.72		3.56	5.86	
	MIN	2.57	3.66		3.66	4.4	
	MAX	19.86	37.06		19.37	37.06	
	95% CI	7.42–8.9	10.64–12.78		7.21–9.54	10.97–14.51	
PESI	Mean	91.14	133	<0.01	93.05	147.61	<0.01
	StdDev	32.72	44.6		34.61	46.74	
	IQR	46.5	73.25		42	81	
	MIN	65	21		21	65	
	MAX	247	166		207	247	
	95% CI	83.86–98.42	123.09–142.94		82.26–103.83	132.86–162.36	
SPESI	Mean	1.15	1.96	<0.01	1.12	1.9	<0.01
	StdDev	1.06	1.23		1.09	1.31	
	IQR	2	2		2	2	
	MIN	0	0		0	0	
	MAX	5	4		4	5	
	95% CI	0.91–1.39	1.69–2.24		0.78–1.46	1.9–2.73	

CBC < 24 h—group with first available CBC under 24 h; NLR—neutrophil-to-lymphocyte ratio; StdDev—standard deviation; IQR—interquartile range; MIN—minimum observed value; MAX—maximum observed value; 95%CI—95% confidence interval for the mean; WBC—white blood cell; PESI—pulmonary embolism severity index; SPESI—simplified pulmonary embolism severity index.

**Table 5:** Patient characteristics across NLR categories (categorical variables, % of column categories).

Variable	Values	Whole Group			CBC < 24 h		
		Elevated NLR (>3.7)		*p*-Value	Elevated NLR (>4.69)		*p*-Value
		No	Yes		No	Yes	
COVID-19	No	78 (54.2%)	66 (45.8%)	<0.01	41 (55.4%)	33 (44.6%)	0.015
	Yes	2 (12.5%)	14 (87.5%)		1 (11.1%)	8 (88.9%)	
Cancer	No	63 (55.3%)	51 (44.7%)	0.036	29 (52.7%)	26 (47.3%)	0.587
	Yes	17 (37%)	29 (63%)		13 (46.4%)	15 (53.6%)	
Chronic heart failure	No	59 (51.8%)	55 (48.2%)	0.485	31 (52.5%)	28 (47.5%)	0.579
	Yes	21 (45.7%)	25 (54.3%)		11 (45.8%)	13 (54.2%)	
Chronic pulmonary disease	No	63 (52.5%)	57 (47.5%)	0.273	31 (51.7%)	29 (48.3%)	0.754
	Yes	17 (42.5%)	23 (57.5%)		11 (47.8%)	12 (52.2%)	
Pulse rate ≥ 110 b.p.m.	No	66 (52%)	61 (48%)	0.329	36 (57.1%)	27 (42.9%)	0.034
	Yes	14 (42.4%)	19 (57.6%)		6 (30%)	14 (70%)	
Systolic BP < 100 mmHg	No	77 (56.2%)	60 (43.8%)	<0.01	41 (62.1%)	25 (37.9%)	<0.01
	Yes	3 (13%)	20 (87%)		1 (5.9%)	16 (94.1%)	
Respiratory rate > 30 breaths/min	No	77 (51.7%)	72 (48.3%)	0.118	39 (52%)	36 (48%)	0.483
	Yes	3 (27.3%)	8 (72.7%)		3 (37.5%)	5 (62.5%)	
Altered mental status	No	77 (57.5%)	57 (42.5%)	<0.01	40 (60.6%)	26 (39.4%)	<0.01
	Yes	3 (11.5%)	23 (88.5%)		2 (11.8%)	15 (88.2%)	
Arterial oxyhemoglobin Saturation < 90%	No	65 (59.6%)	44 (40.4%)	<0.01	37 (68.5%)	17 (31.5%)	<0.01
	Yes	15 (29.4%)	36 (70.6%)		5 (17.2%)	24 (82.8%)	
Concomitant DVT	No	48 (48%)	52 (52%)	0.514	28 (50.9%)	27 (49.1%)	0.938
	Yes	32 (53.3%)	28 (46.7%)		14 (50%)	14 (50%)	
SPESI risk category	Low	28 (76.5%)	8 (23.5%)	<0.01	13 (81.3%)	3 (18.8%)	<0.01
	High	54 (42.9%)	72 (57.1%)		29 (43.3%)	38 (56.7%)	
PESI risk category	Very low	16 (94.1%)	1 (5.9%)	<0.01	8 (88.9%)	1 (11.1%)	<0.01
	Low	20 (71.4%)	8 (28.6%)		10 (76.9%)	3 (23.1%)	
	Intermediate	18 (47.4%)	20 (52.6%)		10 (58.8%)	7 (41.2%)	
	High risk	16 (57.1%)	12 (42.9%)		10 (83.3%)	2 (16.7%)	
	Very high	10 (20.4%)	39 (79.6%)		4 (12.5%)	28 (87.5%)	
Dilated RV	No	39 (52%)	36 (48%)	0.635	18 (47.4%)	20 (52.6%)	0.662
	Yes	41 (48.2%)	44 (51.8%)		24 (53.3%)	21 (46.7%)	
RV dysfunction	No	75 (48.7%)	79 (51.3%)	0.210	40 (50%)	40 (50%)	1
	Yes	5 (83.3%)	1 (16.7%)		2 (66.7%)	1 (33.3%)	
Dilated VCI	No	70 (47.9%)	76 (52.1%)	0.093	38 (49.4%)	39 (50.6%)	0.676
	Yes	10 (71.4%)	4 (28.6%)		4 (66.7%)	2 (33.3%)	
Intracavitary thrombus	No	80 (51.6%)	75 (48.4%)	0.059	42 (51.9%)	39 (48.1%)	0.241
	Yes	0 (0%)	5 (100%)		0 (0%)	2 (100%)	
Leukocytosis	No	66 (68%)	31 (32%)	<0.01	33 (71.7%)	13 (28.3%)	<0.01
	Yes	14 (22.2%)	49 (77.8%)		9 (24.3%)	28 (75.7%)	
In-hospital death	No	79 (59%)	55 (41%)	<0.01	40 (60.6%)	26 (39.4%)	<0.01
	Yes	1 (3.8%)	25 (96.2%)		2 (11.8%)	15 (88.2%)	

CBC < 24 h—group with first available CBC under 24 h; NLR—neutrophil-to-lymphocyte ratio; BP—blood pressure; NLR—neutrophil-to-lymphocyte ratio; DVT—deep vein thrombosis; RV—right ventricle; VCI—vena cava inferior.

**Table 6:** Numerical variables and in-hospital mortality.

Variable	Descriptive Parameter	Whole Group			CBC < 24 h		
		In Hospital Death		*p*-Value	In Hospital Death		*p*-Value
		No	Yes		No	Yes	
Age	Mean	65.51	70.96	0.143	63.91	70.47	0.168
	StdDev	15.61	12.83		16.59	15.19	
	IQR	17	17		20	20	
	MIN	21	42		21	42	
	MAX	94	93		94	93	
	95% CI	62.84–68.17	65.78–76.15		59.83–67.99	62.66–78.28	
WBC Count (10^3^/µL)	Mean	9.29	13.24	<0.01	9.84	13.24	0.136
	StdDev	3.66	6.58		3.97	8.04	
	IQR	5.08	6.96		5.87	9.37	
	MIN	2.57	4.4		3.66	4.4	
	MAX	20.52	37.06		20.52	37.06	
	95% CI	8.67–9.92	10.58–15.9		8.86–10.81	9.1–17.37	
NLR	Mean	4.77	14.91	<0.01	5.59	15.57	<0.01
	StdDev	4.11	12.36		4.6	13.22	
	IQR	3.11	13.29		5.45	11.35	
	MIN	0.3	2.80		0.45	2.8	
	MAX	20.27	45.87		19.99	45.87	
	95% CI	4.07–5.47	9.91–19.90		4.46–6.72	8.78–22.37	
PESI	Mean	103.11	158.26	<0.01	106.85	171.06	<0.01
	StdDev	37.44	48.61		41.28	44.57	
	IQR	41.75	85.75		52.25	70.5	
	MIN	21	66		21	77	
	MAX	209	247		209	247	
	95% CI	96.71–109.51	138.63–177.9		96.7–117	148.15–193.97	
SPESI	Mean	1.34	2.69	<0.01	1.39	2.94	<0.01
	StdDev	1.12	1.01		1.25	0.9	
	IQR	1	1		1	2	
	MIN	0	0		0	1	
	MAX	5	4		5	4	
	95% CI	1.14–1.53	2.28–3.10		1.09–1.7	2.48–3.40	

CBC < 24 h—group with first available CBC under 24 h; NLR—neutrophil-to-lymphocyte ratio; StdDev—standard deviation; IQR—interquartile range; MIN—minimum observed value; MAX—maximum observed value; 95%CI—95% confidence interval for the mean; WBC—white blood cell; PESI—pulmonary embolism severity index; SPESI—simplified pulmonary embolism severity index.

**Table 7:** Categorical variables and in-hospital mortality (% of column categories).

Variable	Values	Whole Group			CBC < 24 h		
		In-Hospital Death		*p*-Value	In-Hospital Death		*p*-Value
		No	Yes		No	Yes	
COVID-19	No	127 (88.2%)	17 (11.8%)	<0.01	63 (85.1%)	11 (14.9%)	<0.01
	Yes	7 (43.8%)	9 (56.3%)		3 (33.3%)	6 (66.7%)	
Cancer	No	95 (83.3%)	19 (16.7%)	0.822	44 (80%)	11 (20%)	1
	Yes	39 (84.8%)	7 (15.2%)		22 (78.6%)	6 (21.4%)	
Chronic heart failure	No	100 (87.7%)	14 (12.3%)	0.032	49 (83.1%)	10 (16.9%)	0.239
	Yes	34 (73.9%)	12 26.1%)		17 (70.8%)	7 (29.2%)	
Chronic pulmonary disease	No	105 (87.5%)	15 (12.5%)	0.026	50 (83.3%)	10 (16.7%)	0.224
	Yes	29 (72.5%)	11 (27.5%)		16 (69.6%)	7 (30.4%)	
Pulse rate ≥ 110 b.p.m.	No	109 (85.8%)	18 (14.2%)	0.162	52 (82.5%)	11 (17.5%)	0.339
	Yes	25 (75.8%)	8 (24.2%)		14 (70%)	6 (30%)	
Systolic BP < 100 mmHg	No	120 (87.6%)	17 (12.4%)	<0.01	56 (84.8%)	10 (15.2%)	0.038
	Yes	14 (60.9%)	9 (31.9%)		10 (58.8%)	7 (41.2%)	
Respiratory rate > 30 breaths/min	No	128 (85.9%)	21 (14.1%)	0.018	62 (82.7%)	13 (17.3%)	0.051
	Yes	6 (54.5%)	5 (45.5%)		4 (50%)	4 (50%)	
Altered mental status	No	121 (90.3%)	13 (9.7%)	<0.01	58 (87.9%)	8 (12.1%)	<0.01
	Yes	13 (50%)	13 (50%)		8 (47.1%)	9 (52.9%)	
Arterial oxyhemoglobin Saturation < 90%	No	103 (94.5%)	6 (5.5%)	<0.01	52 (96.3%)	2 (3.7%)	<0.01
	Yes	31 (60.8%)	20 (39.2%)		14 (48.3%)	15 (51.7%)	
Concomitant DVT	No	81 (81%)	19 (9%)	0.223	43 (78.2%)	12 (21.8%)	0.672
	Yes	53 (88.3%)	7 (11.7%)		23 (82.1%)	5 (17.9%)	
SPESI risk category	Low	33 (97.1%)	1 (2.9%)	0.018	16 (100%)	0 (0%)	0.034
	High	101 (80.2%)	25 (19.8%)		50 (74.6%)	17 (25.4%)	
PESI risk category	Very low	17 (100%)	0 (0%)	<0.01	9 (100%)	0 (0%)	<0.01
	Low	26 (92.9%)	2 (7.1%)		12 (92.3%)	1 (7.7%)	
	Intermediate	36 (94.7%)	2 (5.3%)		17 (100%)	0 (0%)	
	High risk	25 (89.3%)	3 (10.7%)		11 (91.7%)	1 (8.3%)	
	Very high	30 (61.2%)	19 (38.8%)		17 (53.1%)	15 (46.9%)	
Dilated RV	No	62 (82.7%)	13 (17.3%)	0.727	28 (73.7%)	10 (26.3%)	0.226
	Yes	72 (84.7%)	13 (15.3%)		38 (84.8%)	7 (15.6%)	
RV dysfunction	No	128 (83.1%)	26 (16.9%)	0.59	63 (78.8%)	17 (21.3%)	1
	Yes	6 (100%)	0 (0%)		3 (100%)	0 (0%)	
Dilated VCI	No	120 (82.2%)	26 (17.8%)	0,129	60 (77.9%)	17 (22.1%)	0.338
	Yes	14 (100%)	0 (0%)		6 (100%)	0 (0%)	
Intracavitary thrombus	No	130 (83.9%)	25 (16.1%)	1	65 (80.2%)	16 (19.8%)	0.37
	Yes	4 (80%)	1 (20%)		1 (50%)	1 (50%)	
Leukocytosis	No	87 (89.7%)	10 (10.3%)	0.11	38 (82.6%)	8 (17.4%)	0.437
	Yes	47 (74.6%)	16 (25.4%)		28 (75.7%)	9 (24.3%)	

CBC < 24 h—group with first available CBC under 24 h; BP—blood pressure; NLR—neutrophil-to-lymphocyte ratio; DVT—deep vein thrombosis; RV—right ventricle; VCI—vena cava inferior.

**Table 8:** Binary regression model.

Variable	β	*p*	BCa 95% CI for β	
			Lower	Higher
COVID-19	1.68	<0.01	0.01	19.58
Altered mental status	1.56	<0.01	0.2	3.35
Arterial oxyhemoglobin Saturation < 90%	1.98	<0.01	0.27	35.77
Elevated NLR (>3.7)	2.71	0.016	0.59	20.66
Constant	−5.43	<0.01	−22.71	−4.43

NLR—neutrophil-to-lymphocyte ratio.

**Table 9:** Area under ROC curves for numerical variables.

Variable	Area under ROC Curve
NLR	0.853
WBC Count	0.714
SPESI	0.814
PESI	0.812

NLR—neutrophil-to-lymphocyte ratio; WBC—white blood cell; PESI—pulmonary embolism severity index; SPESI—simplified pulmonary embolism severity index.

**Table 10:** Area under ROC curves for numerical variables (<24 h CBC subanalysis).

Variable	Area under ROC Curve
NLR	0.812
WBC Count	0.618
SPESI	0.837
PESI	0.856

NLR—neutrophil-to-lymphocyte ratio; WBC—white blood cell; PESI—pulmonary embolism severity index; SPESI—simplified pulmonary embolism severity index.

**Table 11:** Two-step cluster analysis results.

Variable	Category	Cluster 1	Cluster 2	Cluster 3	Cluster 4	Cluster 5	Predictor Importance
Count (% of total)	-	26 (16.2%)	52 (32.5%)	8 (5%)	59 (36.9%)	15 (9.4%)	-
SPESI category	Low risk	26 (100%)	0 (0%)	8 (100%)	0 (0%)	0 (0%)	1.0
	High risk	0 (0%)	52 (100%)	0 (0%)	59 (100%)	15 (100%)	
Elevated NLR (>3.7)	No	26 (100%)	52 (100%)	0 (0%)	0 (0%)	2 (13.3%)	0.95
	Yes	0 (0%)	0 (0%)	8 (100%)	59 (100%)	13 (86.7%)	
COVID-19	No	26 (100%)	52 (100%)	7 (87.5%)	59 (100%)	0 (0%)	0.94
	Yes	0 (0%)	0 (0%)	1 (12.5%)	0 (0%)	15 (100%)	

NLR—neutrophil-to-lymphocyte ratio; SPESI—simplified pulmonary embolism severity index.

**Table 12:** Two-step cluster analysis results.

Variable	Category	Cluster 1a	Cluster 2a	Cluster 3a	Cluster 4a	Predictor Importance
Count (% of total)	-	16 (19.3%)	28 (33.7%)	30 (36.1%)	9 (10.8%)	-
sPESI category	Low-risk	16 (100%)	0 (0%)	0 (0%)	0 (0%)	1
	High-risk	0 (0%)	28 (100%)	30 (100%)	9 (100%)	
Elevated NLR (>4.69)	No	13 (81.3%)	28 (100%)	0 (0%)	1 (11.1%)	0.83
	Yes	3 (18.8%)	0 (0%)	30 (100%)	8 (88.9%)	
COVID-19	No	16 (100%)	28 (100%)	30 (100%)	0 (0%)	1
	Yes	0 (0%)	0 (0%)	0 (0%)	9 (100%)	

NLR—neutrophil-to-lymphocyte ratio; SPESI—simplified pulmonary embolism severity index.

## Data Availability

The data presented in this study are available upon reasonable request from the corresponding author.
